# Delayed presentation of an apical-third horizontal root fracture: conservative endodontic management and aesthetic rehabilitation — a case report

**DOI:** 10.3389/fdmed.2026.1833303

**Published:** 2026-07-15

**Authors:** Monika Kumal, Regan Pokharel, Dinesh Rokaya, Ahmad Al Jaghsi, Shantanu Dixit, Ashok Ayer

**Affiliations:** 1 People's Dental College and Hospital, Kathmandu, Nepal; 2 Department of Conservative Dentistry and Endodontics, People's Dental College and Hospital, Kathmandu, Nepal; 3 Clinical Sciences Department, College of Dentistry, Ajman University, Ajman, United Arab Emirates; 4 Center of Medical and Bio-Allied Health Sciences Research, Ajman University, Ajman, United Arab Emirates; 5 Department of Prosthodontics, Gerodontology, and Dental Materials, Greifswald University Medicine, Greifswald, Germany; 6 Dental Imaging & Diagnostic Center, Ballabgarh, Haryana, India; 7 Department of Conservative Dentistry and Endodontics, B.P. Koirala Institute of Health Sciences, Dharan, Nepal

**Keywords:** apical-third root fracture, bioceramic sealer, delayed management, dental trauma, lithium disilicate veneer, pulp necrosis

## Abstract

Horizontal root fractures represent an uncommon form of dental trauma, accounting for approximately 0.5%–7% of all injuries to permanent teeth. Pulp necrosis occurs in nearly 25% of overall root fractures, most frequently within the first few months after injury. Outcomes in cases presenting long after trauma, however, are not well documented. A 33-year-old female sought care for discoloration of the maxillary left incisors one year after sustaining dental trauma. Clinical findings, supported by radiographic assessment, confirmed pulpal necrosis in teeth 21 and 22. An apical-third horizontal root fracture was identified in tooth 22. Endodontic management was limited to the coronal fragment, using a calcium silicate–based bioceramic sealer, with the apical segment left undisturbed. Following endodontic stabilization, aesthetic rehabilitation was achieved with lithium disilicate veneers extending from teeth 11 to 23. At the 12-month review, radiographic evaluation demonstrated hard-tissue repair at the fracture interface and evidence of gradual apical fragment resorption. The aesthetic result remained stable. This case underscores that delayed presentation of apical-third root fractures does not necessarily compromise healing. When biological principles are respected and treatment is confined to the necrotic coronal segment, preservation of the vital apical fragment can still yield favorable outcomes**.**

## 1 Introduction

Traumatic dental injuries affect approximately 20%–30% of individuals worldwide and are associated with significant functional, aesthetic, and economic consequences (1). Among these injuries, horizontal root fractures involving dentin, cementum, and pulp account for 0.5%–7% of traumatic injuries to permanent teeth (2, 3).

Horizontal root fractures are seen most often in adolescents and young adults, with maxillary incisors particularly vulnerable because of their prominent position and exposure to frontal impact (2, 4). Based on the location of the fracture line, these injuries are classified as cervical, middle, or apical third fractures (4, 5). Middle-third fractures are reported most frequently, whereas apical and cervical third fractures occur with nearly similar incidence (1).

Apical-third fractures generally have the most favourable prognosis because the apical fragment often remains vital (2, 3). Treatment decisions depend on several factors, including fracture level, displacement of the coronal fragment, and pulp status (5). When pulpal necrosis develops, endodontic therapy is indicated but is typically limited to the coronal fragment, preserving the vitality of the apical portion (2, 6).

Trauma-induced pulp necrosis most commonly becomes evident within the first 3–4 months after injury (1, 5). However, information regarding outcomes in patients presenting long after this period is scarce. A recent cohort study showed that timely management is associated with favourable healing but did not specifically evaluate cases with very late presentation (7).

In addition to biological considerations, trauma to anterior teeth frequently results in discolouration, structural compromise, or spacing concerns that may require aesthetic rehabilitation. Post-endodontic restorations such as veneers or crowns can restore appearance while reinforcing weakened tooth structure (1).

This raises an important clinical question: can treatment principles established for early intervention—particularly the preservation of a potentially vital apical fragment and restriction of endodontic therapy to the infected coronal segment—still be effective when diagnosis is significantly delayed after the initial traumatic event? Delayed presentation may be associated with altered pulpal status, progressive reparative changes at the fracture interface, or diagnostic uncertainty, all of which can complicate treatment planning and prognosis assessment. The present report describes the management of an apical-third horizontal root fracture diagnosed 12 months after trauma, a timeframe in which spontaneous healing or stabilization may already have occurred. Despite this delayed diagnosis, conservative management was undertaken, and follow-up demonstrated uneventful healing, preservation of function, and successful aesthetic rehabilitation using porcelain veneers. This case therefore highlights the potential for favorable outcomes even in delayed presentations when biologically based conservative principles are appropriately applied, although such findings should be interpreted cautiously given the limited evidence from single-case observations.

## 2 Case presentation

### 2.1 Timeline

A summary of the sequence of treatment and follow-up is provided in Table 1.

**Table 1:** Timeline of treatment and follow-up.

Time point	Clinical procedure
Initial visit	Clinical and radiographic examination; diagnosis established.
First appointment	Initiation of root canal treatment; intracanal medicament placed.
Second appointment	Obturation and coronal restoration. completed.
Third appointment	Tooth preparation for veneers and digital impression.
Fourth appointment	Try-in and definitive cementation of veneers.
12-month follow-up	Clinical and radiographic review.

### 2.2 Clinical and radiological evaluation

A 33-year-old female reported to the Department of Conservative Dentistry and Endodontics with a complaint of fractured upper front teeth following trauma that had occurred approximately 12 months earlier. Her medical history was non-contributory, and she did not report any drug allergies.

Extraoral examination did not reveal any abnormalities. Intraoral examination showed generalized diastemata involving both maxillary and mandibular anterior teeth. The maxillary left central and lateral incisors (FDI 21 and 22) appeared noticeably discolored (Figure 1A). Both teeth were firm, with normal periodontal status (probing depths ≤3 mm and no bleeding on probing).

**Figure 1:**
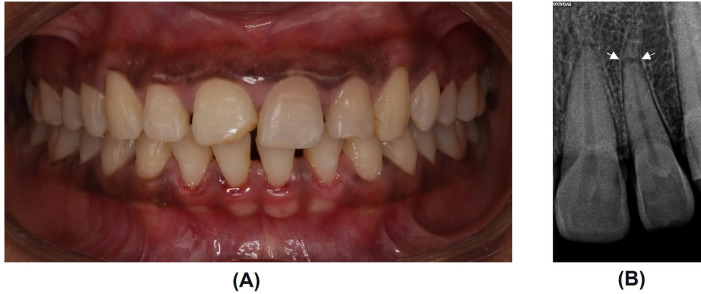
Pre-operative clinical and radiographic presentation. **(A)** Intraoral photograph showing generalized diastemata and discoloration of the maxillary left central and lateral incisors (teeth 21 and 22). **(B)** Periapical radiograph of teeth 21 and 22 demonstrating a horizontal radiolucent fracture line at the apical third of tooth 22 (arrow). Widening of the periodontal ligament space is evident in teeth 21 and 22. No periapical radiolucency or root resorption is observed.

Pulp sensibility testing with cold spray (Endo-Ice, Coltene) elicited no response from teeth 21 and 22, while adjacent teeth (11, 12, 13, and 23) responded within normal limits. Ellis Class I fractures were observed in teeth 11 and 23, whereas teeth 21 and 22 showed features of complicated crown fractures. The surrounding gingival tissues appeared healthy, with no evidence of soft-tissue injury. Although tooth 11 presented with an oblique enamel-dentin fracture, clinical and radiographic evaluation demonstrated no signs of pulpal or periodontal injury.

Intraoral periapical radiography demonstrated widening of the periodontal ligament space in teeth 21 and 22. A distinct radiolucent horizontal line was visible at the apical third of the root of tooth 22, consistent with a fracture involving cementum, dentin, and pulp (Figure 1B). No periapical radiolucency or root resorption was detected in the neighboring teeth.

Based on these findings, a diagnosis of pulpal necrosis in teeth 21 and 22 secondary to trauma was made, along with an apical-third horizontal root fracture in tooth 22.

### 2.3 Endodontic management

Root canal treatment was planned for teeth 21 and 22. Instrumentation was confined to the coronal segment up to the fracture line to preserve the vitality of the apical fragment.

After achieving rubber dam isolation and administering local anaesthesia (2% lidocaine with 1:100,000 adrenaline), access cavities were prepared using a #2 Endo-Access bur (Maillefer/Dentsply, Switzerland) under 3.5× magnification dental loupes (Figure 2A). Working length was determined with an electronic apex locator (Root ZX Mini, J Morita, Kyoto, Japan) (Figure 2B).

**Figure 2:**
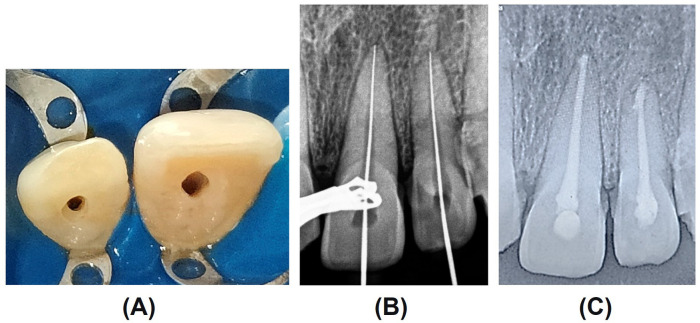
Endodontic management confined to the coronal fragment. **(A)** Access cavity preparation in teeth 21 and 22 under rubber dam isolation. **(B)** Working length determination radiograph. **(C)** Post-obturation radiograph showing root canal filling to the radiographic apex in tooth 21 and termination of the obturation at the fracture line in tooth 22 (arrow), preserving the apical fragment.

Cleaning and shaping were performed using rotary nickel–titanium instruments (Protaper Universal, Dentsply Maillefer, Ballaigues, Switzerland). Irrigation was carried out with 3% sodium hypochlorite (15 mL per canal), activated with passive ultrasonic irrigation for 30 s (Irrisafe tip, Satelec). A final rinse with 17% EDTA for one minute was followed by saline.

Calcium hydroxide paste (RC Cal, Prime Dental Products Pvt. Ltd., India) was placed as an intracanal medicament, and the patient was recalled after two weeks. At the subsequent visit, master cone fit was verified radiographically, and obturation was completed using a single-cone technique with a calcium silicate-based bioceramic sealer(Ceraseal, Meta Biomed, Chuncheon, Korea) and 25.06% Gutta Percha points (Figure 2C).

The canals were sealed coronally with 4 mm of glass ionomer cement (GC Fuji IX), and the access cavities were restored with composite resin (Beautifil II, Shofu Inc., Japan).

### 2.4 Aesthetic rehabilitation

Because of the existing diastemata, coronal fractures, and the possibility of post-endodontic discoloration, porcelain veneers were selected to restore aesthetics and function for teeth 11, 12, 21, 22, and 23 (Figure 3A).

**Figure 3:**
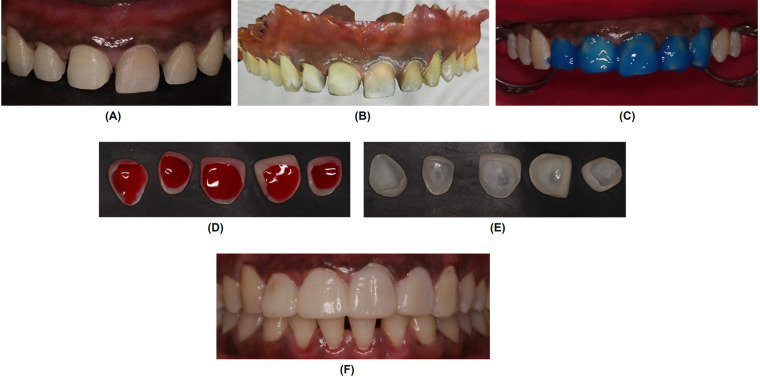
Aesthetic rehabilitation with lithium disilicate veneers. **(A)** Tooth preparation for veneers on teeth 11–23. **(B)** Digital impression obtained using an intraoral scanner. **(C)** Enamel etching with 37% orthophosphoric acid. **(D)** Intaglio surface etching of the veneers with 5% hydrofluoric acid. **(E)** Application of silane coupling agent following rinsing and drying. **(F)** Adhesive cementation of the veneers.

Gingival retraction was achieved using a double-cord technique, beginning with size #00 followed by size #0. A digital impression was obtained using an intraoral scanner (Figure 3B). Provisional restorations were fabricated with a bis-acrylic material (Luxatemp LC, DMG), finished, polished, and cemented with temporary cement.

Vita Shade guide was used for shade selection and patient satisfaction was ensured. Laboratory-fabricated lithium disilicate veneers (e.max, Ivoclar Vivadent) were evaluated at the try-in appointment. Under rubber dam isolation, the tooth surfaces were etched with 37% orthophosphoric acid, rinsed, dried, and treated with a bonding agent (Beautibond, Shofu Inc., Japan) (Figure 3C).

The intaglio surfaces of the veneers were etched with 5% hydrofluoric acid for 20 s, ultrasonically cleaned, dried, and treated with a silane coupling agent (Monobond Plus, Ivoclar) (Figures 3D, E). Cementation was carried out using dual-cure adhesive resin cement (Variolink Esthetic LC, Ivoclar) (Figure 3F).

### 2.5 Follow-Up and outcomes

At the 12-month review, the patient reported no symptoms. Clinical examination showed no tenderness on percussion, mobility, displacement, sinus tract formation, or periodontal abnormalities (Figure 4A). Follow-up intraoral periapical radiography demonstrated formation of a calcified hard-tissue barrier at the fracture site, along with gradual, non-inflammatory remodeling of the apical fragment of tooth 22 consistent with long-term healing. The lamina dura appeared intact, the periodontal ligament space was within normal limits, and no periapical pathology was evident (Figure 4B).

**Figure 4:**
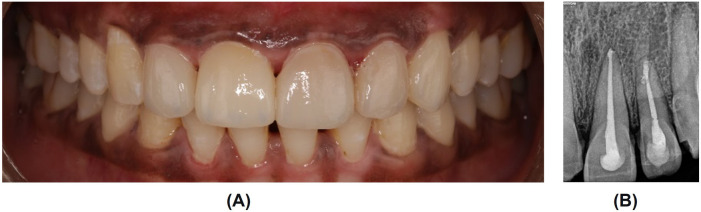
Twelve-month follow-up. **(A)** Intraoral photograph showing healthy gingival tissues, satisfactory marginal adaptation of the veneers, and closure of the diastemata. **(B)** Periapical radiograph demonstrating calcified hard-tissue barrier formation at the fracture site and physiological resorption of the apical fragment (arrows). The lamina dura appears intact, and the periodontal ligament space is within normal limits, with no evidence of periapical pathology.

### 2.6 Patient perspective

The patient reported satisfaction with the treatment outcome, noting resolution of symptoms and restored comfort during function. She was particularly pleased with the improved esthetics following veneer placement, which enhanced her smile and confidence. The patient also expressed initial concern regarding the prognosis due to delayed treatment but felt reassured by the clinical explanations provided throughout management. At follow-up, she remained asymptomatic and satisfied with both functional and esthetic results.

## 3 Discussion

Horizontal root fractures generally have a favourable prognosis, with healing reported in up to 80% of cases even without initial treatment (8, 9). However, the risk of pulp necrosis increases in mature teeth and when the coronal fragment is significantly displaced (8, 10). In the present case, the maxillary lateral incisor (tooth 22) was involved, placing it within the 27% of fractures identified incidentally (11), while maxillary central incisors remain the most commonly affected (68%) (9, 11). The principal finding is that the established treatment principle—confining endodontic intervention to the necrotic coronal fragment while preserving the presumed vital apical fragment—proved effective even with a 12-month delay. This principle rests on the observation that the apical fragment almost always retains vitality (12).

In apical-third fractures, clinical signs are often minimal, and conservative management is usually recommended to allow pulp healing (4, 5). While pulp necrosis typically becomes evident within 3–4 months, it may present much later (5); Çalişkan & Pehlivan (11) documented reversals of vitality up to 2 years post-trauma, consistent with our patient's 12-month presentation. Discoloration and a negative cold test confirmed necrosis—mirroring the case reported by Andrade et al. (9), whose patient also presented at 1 year with crown darkening. Isolated negative responses can be unreliable early after injury (6), but persistent discoloration at 12 months provided definitive evidence, supporting the practice of not relying on pulp testing alone (1). The absence of coronal displacement was critical; Andreasen et al. (10) identified 1 mm diastasis as a threshold beyond which necrosis risk doubles and hard tissue healing becomes unlikely. Andrade et al. (9) further noted that close fragment apposition seals the fracture against oral microorganisms. While a delay of several days does not significantly affect the outcome (12), our case extends this observation to a 12-month interval. A unifying concept from large studies is that healing depends on vascularity at the fracture site (related to age and root development) and the degree of pulp rupture (related to displacement) (10).

Root canal treatment was therefore confined to the coronal fragment, based on the principle that the apical pulp remains vital (5) —a principle established histologically (13) and confirmed clinically (14). In their review of 98 teeth, Cvek et al. (14) found that significantly reduced healing occurred when both fragments were obturated, whereas 76% of apical-third fractures healed with coronal-fragment-only filling, likely facilitated by canal narrowness in this region. Andreasen et al. (12) explained that after coronal necrosis, treatment can yield at best connective tissue union, as the periodontal ligament becomes the dominant healing component. Failure with two-fragment obturation is attributed to overfilling, which may extrude contaminated tissue into the fracture site (14). Obturation with a calcium silicate-based bioceramic sealer (Ceraseal) was chosen for its biocompatibility, alkaline pH, and potential to promote hard-tissue formation (2)—properties paralleling those of calcium hydroxide, which Cvek et al. (14) identified as the treatment of choice. The 12-month radiograph demonstrated calcified hard-tissue barrier formation and progressive peripheral rounding of the apical fragment (Figure 4B), characteristic of healing with calcified tissue (6, 8). No antibiotics were prescribed, in accordance with current evidence and guidelines that do not support their routine use in uncomplicated root fractures (10, 12). Although Andreasen et al. (12) reported an association between antibiotic use and healing outcomes involving granulation tissue, this finding is observational and does not imply a causal relationship.

Following endodontic treatment, the persistent discoloration and pre-existing diastemata necessitated aesthetic rehabilitation. Porcelain veneers were selected to mask discoloration, close spaces, and reinforce tooth structure while preserving healthy tissue (1). The 12-month review confirmed satisfactory aesthetic integration (Figure 4A).

Regular follow-up is essential after root fractures, with reviews at 6 and 12 months and extended monitoring to detect late complications. The present case has a follow-up period of 12 months; however, changes in the apical fragment may continue over time, and in rare instances, late complications such as infection or inflammatory changes may occur. Therefore, a longer follow-up period (≥2–3 years) is required to confirm complete and stable healing. Given the finite longevity of adhesive restorations, longer-term monitoring every 4–5 years is advisable even when healing appears complete (14).

## 4 Conclusion

Delayed diagnosis of an apical-third root fracture does not preclude a favourable outcome when treatment follows established biological principles. Preservation of the vital apical fragment, confinement of endodontic therapy to the coronal segment, and subsequent aesthetic rehabilitation with porcelain veneers resulted in hard-tissue healing at 12 months. This case highlights the importance of clinical vigilance for crown discolouration in patients with remote trauma history and supports the use of biocompatible materials to optimise long-term tooth retention.

## Data Availability

The original contributions presented in the study are included in the article/Supplementary Material, further inquiries can be directed to the corresponding author.
